# High Serum Uric Acid is Highly Associated with a Reduced Left Ventricular Ejection Fraction Rather than Increased Plasma B-type Natriuretic Peptide in Patients with Cardiovascular Diseases

**DOI:** 10.1038/s41598-018-37053-0

**Published:** 2019-01-24

**Authors:** Yoshitsugu Oki, Makoto Kawai, Kosuke Minai, Kazuo Ogawa, Yasunori Inoue, Satoshi Morimoto, Toshikazu Tanaka, Tomohisa Nagoshi, Takayuki Ogawa, Michihiro Yoshimura

**Affiliations:** Division of Cardiology, Department of Internal Medicine, The Jikei University School of Medicine, Tokyo, Japan

## Abstract

High serum uric acid (UA) has been reported to be associated with left ventricular (LV) dysfunction; however, the relationship between UA and plasma B-type natriuretic peptide (BNP), a sensitive biomarker of heart failure, is still unclear. This study investigated their relationship to provide an accurate assessment of high UA. The study patients consisted of 3,077 subjects who underwent cardiac catheterization because of various cardiovascular disorders. Since the explanatory factors of multiple regression analysis were mostly confounding with each other, subgroup analysis was performed by quartering the study population using the respective risk factors and by covariance structure analysis. This analysis revealed that UA was almost always well associated with a reduced LV ejection fraction (LVEF), but generally not with BNP. UA was significantly associated with BNP in lean aged females, but not in obese adolescent males, although LVEF was significantly reduced in response to a high UA in both groups. A high UA is a direct risk factor for cardiac dysfunction from the perspective of BNP; however, augmentation of BNP in response to a high UA would likely be restricted among obese adolescent males. On the other hand, the observed LV systolic dysfunction, such as LVEF, reflects a high UA on an almost constant basis.

## Introduction

High serum uric acid (UA) has recently been discussed not only as a gout culprit but also as a cause of cardiovascular disorders. According to previous studies, serum UA levels predict the progression of chronic kidney disease and the development of stroke^1–3^. Additionally, high serum UA is associated with the presence of hypertension, diabetes, and metabolic syndrome^4–8^. High serum UA levels also predict an increase in morbidity and mortality in patients with heart failure^3,9–13^. Recently, we reported that high UA is causally associated with left ventricular (LV) systolic dysfunction and a reduced LV ejection fraction (LVEF) in patients with ischemic heart disease. Importantly, the effect of high UA on LV dysfunction is exerted not only through an atherosclerotic process in the coronary arteries (cardiac ischemia) but also directly, as represented by a possible cause-and-effect relationship^14^.

B-type natriuretic peptide (BNP) is a natriuretic peptide that is widely used for diagnosis and predicting prognosis in heart failure^15–19^. The plasma level of BNP is a highly reliable surrogate maker of heart failure. Measurement of plasma BNP is recommended as a reliable diagnostic method of heart failure in general practice and emergency medical care^20,21^. It is thus conceivable that plasma BNP should be potentially linked to high serum UA and associated LV dysfunction. There have been few reports of a close linkage between serum UA and plasma BNP in heart failure. Instead, there has been a negative report in which UA did not significantly add to the prognostic utility of BNP^22^. Although a high serum UA level is associated with heart failure, more studies are required to confirm the role of high serum UA in heart failure in consideration of the observed LV dysfunction and plasma BNP.

Statistical analysis would be of assistance in this study; however, analyses are highly intractable for precise study because plasma BNP levels are associated with many confounding factors, including ageing, gender distinction, body mass index (BMI), LV dysfunction, and renal dysfunction^23,24^. Additionally, serum UA is associated with the same factors^14,25^. To explore the precise effects of high UA on plasma BNP and the observed LV dysfunction, advanced statistical procedures are warranted.

In this study, we examined the effect of high serum UA on plasma BNP and observed LV dysfunction in patients with cardiovascular diseases who underwent cardiac catheterization in our institution. We performed a step-by-step statistical analysis to examine the possible action of high serum UA on high plasma BNP and observed LV dysfunction, taking into account possible associated factors such as age, gender distinction, BMI and renal function.

## Results

### Patients’ characteristics

Table 1 shows the patients’ overall and respective sub-group characteristic (gender difference) in this study.

**Table 1:** Characteristics of all patients.

Characteristics	Overall	Female	Male	*P* value
Number (%) or Mean ± SD, Median [interquartile range]				
Numbers of patients	3,077 (100.0)	542 (17.6)	2,535 (82.4)	
Age (years old)	65.7 ± 11.9	69.6 ± 13.0	64.8 ± 11.4	<0.001
BMI (kg/m^2^)	24.4 ± 4.0	22.5 ± 4.5	24.8 ± 3.7	<0.001
Current smoker	651 (21.2)	56 (1.8) (10.3^†^)	595 (19.3) (23.5^†^)	<0.001
Family history of IHD	743 (24.1)	131 (4.3) (24.2^†^)	615 (19.9) (24.1^†^)	0.985
Laboratory findings				
Hb (g/dL)	13.4 ± 1.9	12.2 ± 1.7	13.7 ± 1.8	<0.001
Creatinine (mg/dL)	0.92 ± 0.47	0.76 ± 0.35	0.96 ± 0.49	<0.001
eGFR (mL/min/1.73 m^2^)	68.5 ± 19.6	65.6 ± 20.8	69.1 ± 19.3	<0.001
UA (mg/dL)	6.1 ± 1.5	5.5 ± 1.7	6.2 ± 1.5	<0.001
	6.1 [5.1, 7.0]	5.2 [4.4, 6.5]	6.2 [5.3, 7.1]	
FBS (mg/dL)	121.3 ± 42.4	114.1 ± 32.9	122.9 ± 44.1	<0.001
HbA1c (%)	6.3 ± 1.0	6.1 ± 0.9	6.3 ± 1.0	0.002
TG (mg/dL)	123.4 ± 94.1	105.0 ± 52.5	127.4 ± 100.3	<0.001
HDL-C (mg/dL)	51.7 ± 15.3	58.8 ± 16.6	50.2 ± 14.5	<0.001
LDL-C (mg/dL)	101.6 ± 30.4	107.8 ± 32.0	100.2 ± 29.9	<0.001
LDL-C/HDL-C	2.1 ± 0.8	2.0 ± 0.8	2.1 ± 0.8	<0.001
CRP (mg/dL)	0.46 ± 1.36	0.48 ± 1.47	0.46 ± 1.34	0.722
BNP (ng/L)	122.5 ± 261.1	181.9 ± 351.9	109.7 ± 235.2	<0.001
	43.2 [16.7, 123.3]	70.0 [26.3, 192.6]	38.6 [15.2, 111.0]	
Left ventricular hemodynamic parameter				
LVEF (%)	56.9 ± 11.8	59.0 ± 11.7	56.4 ± 11.8	<0.001
Underlying cardiovascular disease				
Ischemic heart disease	2591 (84.2)	375 (12.2) (69.2^†^)	2216 (72.0) (87.4^†^)	<0.001
Acute coronary syndrome	444 (14.4)	69 (2.2) (12.7^†^)	375 (12.2) (14.8^†^)	0.215
Angina pectoris	2154 (70.0)	287 (9.3) (53.0^†^)	1867 (60.7) (73.6^†^)	<0.001
Cardiomyopathy	236 (7.7)	62 (2.0) (11.4^†^)	174 (5.7) (6.9^†^)	<0.001
Valvular disease	220 (7.1)	77 (2.5) (14.2^†^)	143 (4.6) (5.6^†^)	<0.001
Arrhythmia	416 (13.5)	80 (2.7) (14.8^†^)	336 (10.9) (13.3^†^)	0.352
Atrial fibrillation	190 (6.2)	38 (1.3) (7.0^†^)	152 (4.9) (6.0^†^)	0.327
Other than AF	226 (7.3)	42 (1.4) (7.7^†^)	184 (6.0) (7.3^†^)	0.691
Other clinical diagnosis				
Hypertension	2279 (74.1)	373 (12.1) (68.8^†^)	1906 (61.9) (75.2^†^)	0.002
Type-2 diabetes mellitus	1212 (39.4)	175 (5.7) (32.3^†^)	1037 (33.7) (40.9^†^)	<0.001
Dyslipidemia	2221 (72.2)	368 (12.0) (67.9^†^)	1853 (60.2) (73.1^†^)	0.014
Medication				
Antiplatelet agents	2153 (70.0)	299 (9.7) (55.2^†^)	1854 (60.3) (73.1^†^)	<0.001
Anticoagulant agents	407 (13.2)	79 (2.6) (14.6^†^)	328 (10.7) (12.9^†^)	0.307
ACE inhibitors	597 (19.4)	82 (2.7) (15.1^†^)	515 (16.7) (20.3^†^)	0.006
ARBs	1201 (39.0)	202 (6.6) (37.3^†^)	999 (32.5) (39.4^†^)	0.354
Beta blockers	1235 (40.1)	187 (6.1) (34.5^†^)	1048 (34.1) (41.3^†^)	0.003
Calcium channel blockers	1626 (52.8)	270 (8.8) (49.8^†^)	1356 (44.1) (53.5^†^)	0.120
Diuretics	633 (20.6)	144 (4.7) (26.6^†^)	489 (15.9) (19.3^†^)	<0.001
Anti-hyperuricemia	501 (16.3)	35 (1.1) (6.5^†^)	466 (15.1) (18.4^†^)	<0.001
Statins	1794 (58.3)	291 (9.5) (53.7^†^)	1503 (48.8) (59.3^†^)	0.016
Other than statins	349 (11.3)	65 (2.1) (12.0^†^)	284 (9.2) (11.2^†^)	0.599
Oral antidiabetic agents	839 (27.3)	120 (3.9) (22.1^†^)	719 (23.4) (28.4^†^)	0.003
Insulin	276 (9.0)	50 (1.6) (9.2^†^)	236 (7.3) (8.9^†^)	0.819

Hb, hemoglobin; UA, uric acid; FBS, fasting blood sugar; HbA1c, hemoglobin A1c; TG, triglycerides; HDL-C, high-density lipoprotein cholesterol; LDL-C, low-density lipoprotein cholesterol; ACE, angiotensin-converting enzyme; ARBs, angiotensin II type I-receptor blockers; BMI, body mass index; BNP, B-type natriuretic peptide; CRP, C-reactive protein; eGFR, estimated glomerular filtration rate; IHD, ischemic heart disease; and LVEF, left ventricular ejection fraction. ^†^, the percentage of each sub-group of patients. The significant difference comparisons between female group and male group were described using *P* value.

### Single regression analysis among serum UA, plasma BNP and LVEF

**Figure 1:**
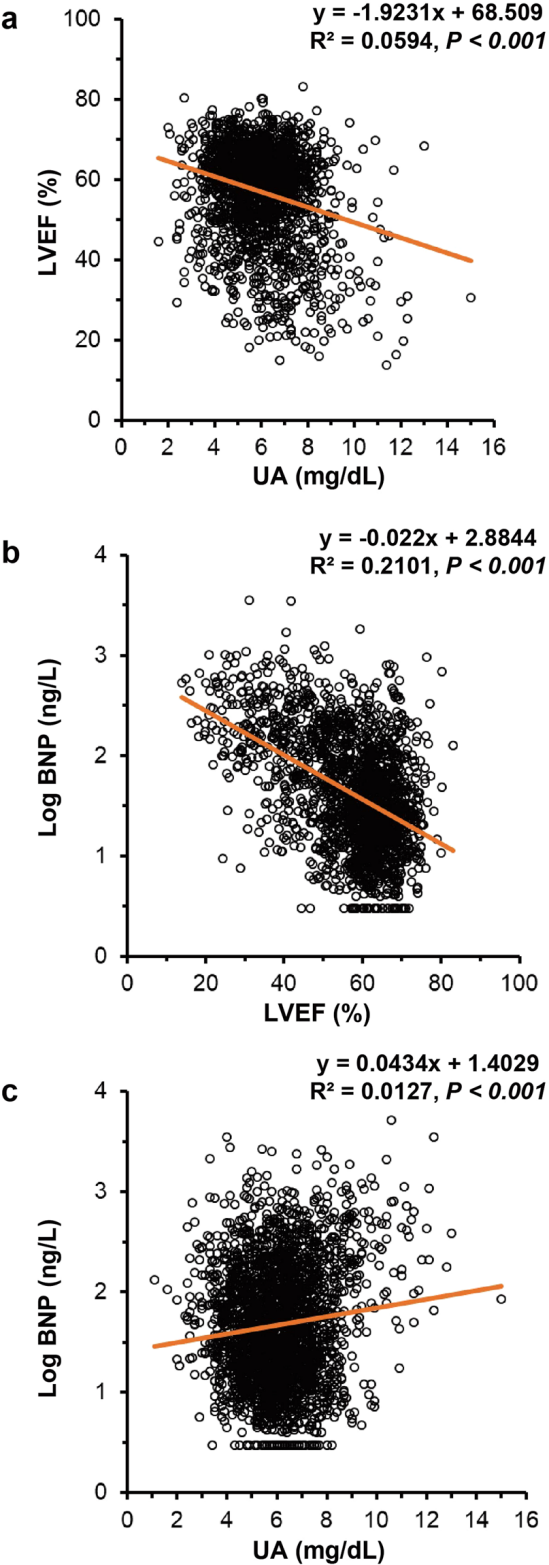
Single regression analysis among serum UA, plasma BNP and LVEF Scatter plots and a simple regression line (orange straight line) with the regression equation demonstrate the correlation between two values among UA, plasma BNP, and LVEF. BNP, B-type natriuretic peptide; LVEF, left ventricular ejection fraction; UA, uric acid.

Figure 1 shows the single regression analysis between two values among UA, plasma BNP, and LVEF. There was a significant correlation in the respective graphs (*P* < 0.001). There appears to be a close linkage among serum UA, plasma BNP and LVEF.

### Single regression analysis among serum UA, plasma BNP, LVEF and other risk factors

**Figure 2:**
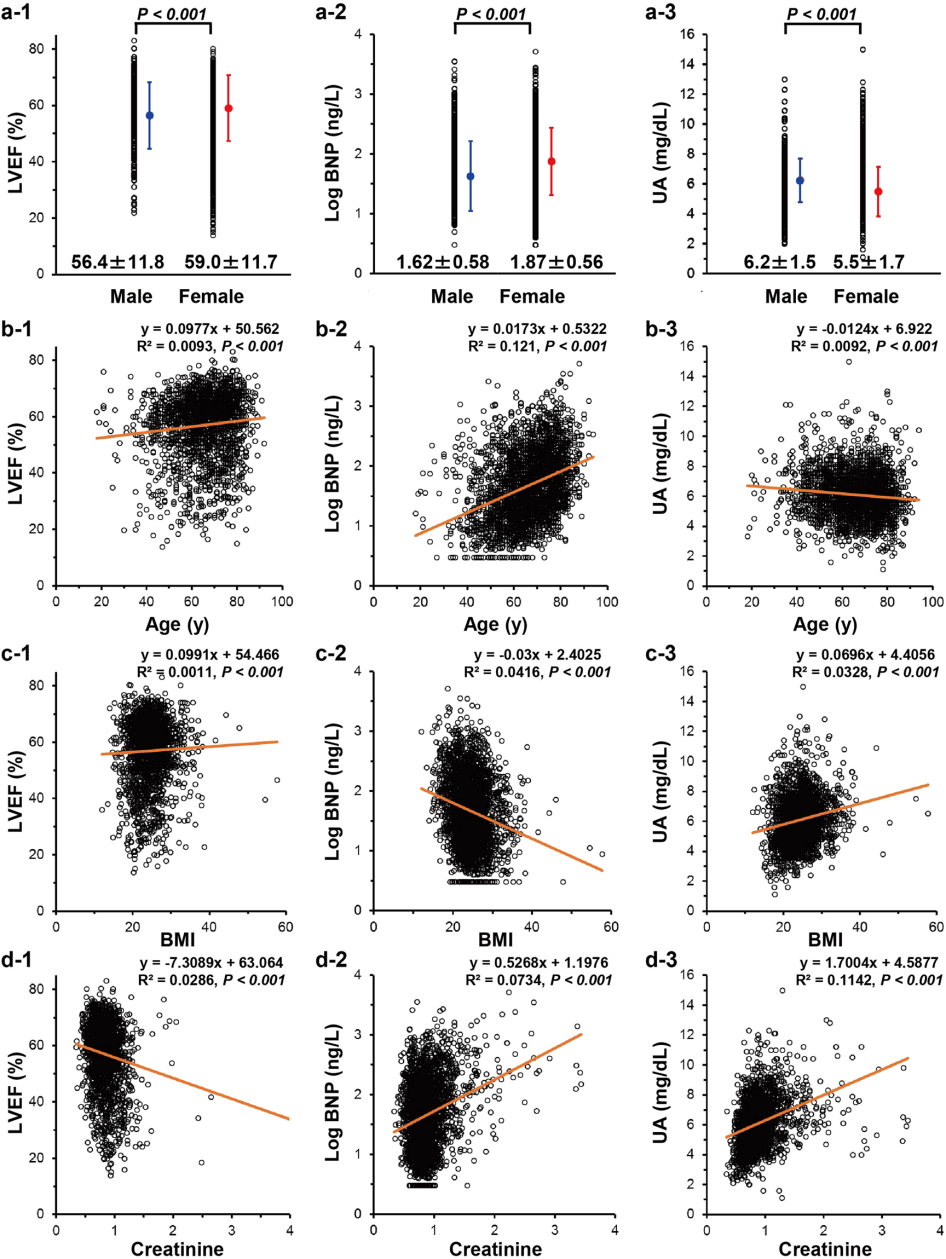
Single regression analysis among serum UA, plasma BNP, LVEF and other risk factors Scatter plots and a simple regression line (orange straight line) with the regression equation demonstrate the correlation between two values among UA, plasma BNP, LVEF, gender, age, BMI, and creatinine. The means of each value are marked in blue (male) or red (female) plots in each panel, with standard deviations (same colored lines). Statistical significance was indicated as the *P* value with the horizontal bar in the upper part of each scatter plot in the top three panels. BNP, B-type natriuretic peptide; LVEF, left ventricular ejection fraction; UA, uric acid.

**Figure 3:**
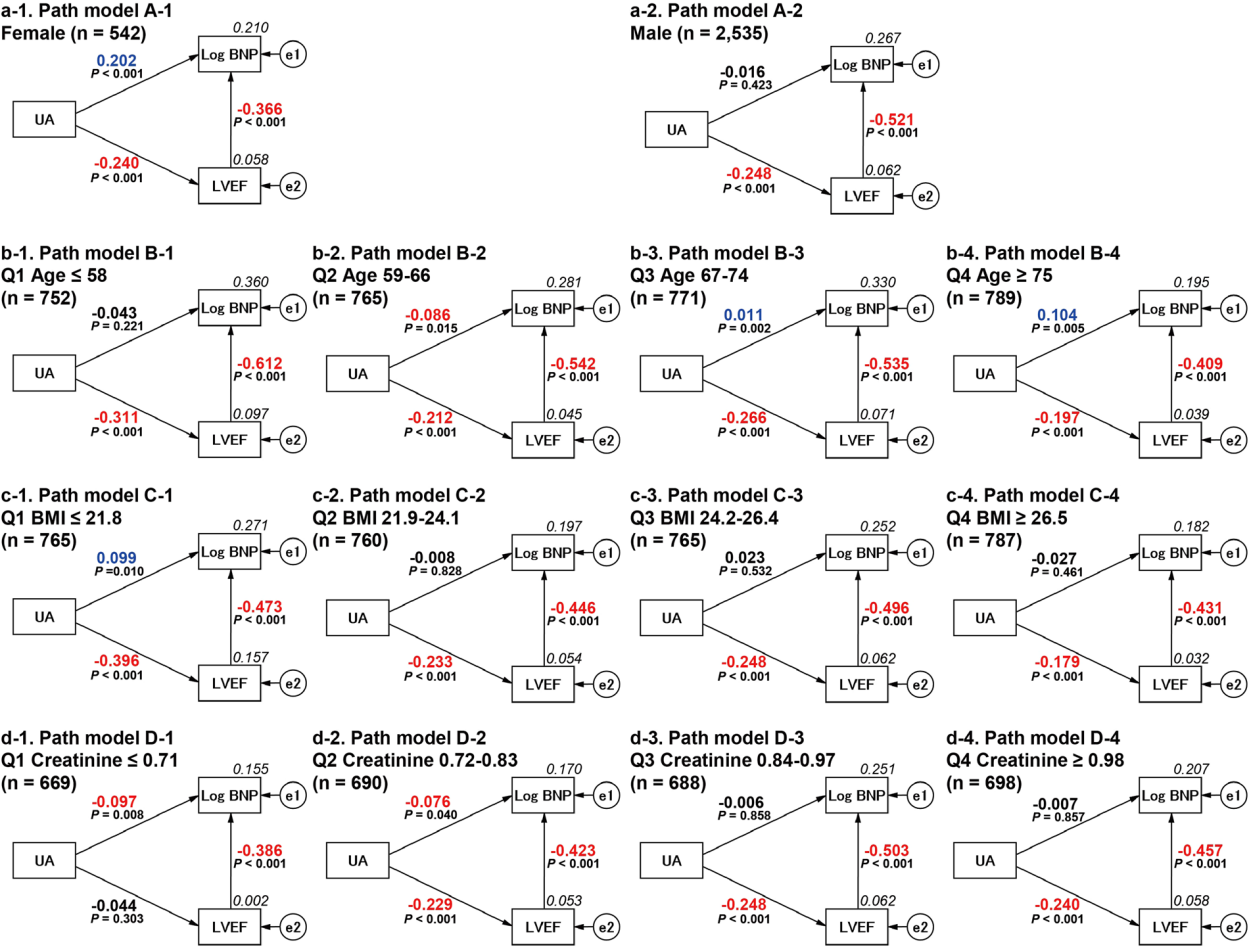
Path models from A-1 to D-4 were dependent on the gender difference, ageing, BMI, and creatinine (the groups quartered by each value) The path has a coefficient showing the standardized coefficient of a regressing independent variable on a dependent variable of the relevant path (UA to Log BNP; UA to LVEF; LVEF to Log BNP). These variables are standardized regression coefficients (direct effect) with *P* values if statistical significance was present, squared multiple correlations [on the upper right side of rectangles (in italics)] and correlations among exogenous variables. The positive regression coefficients are indicated as blue-colored values, and the negative regression coefficients are indicated as red-colored values. Uncolored values denote *P* values less than 0.05. BNP, B-type natriuretic peptide; LVEF, left ventricular ejection fraction; UA, uric acid; e, extraneous variable.

Several potential risk factors, which were listed as candidates based on previous information, were included in the analysis: gender, age, BMI and creatinine (Cr). To examine a possible interruption of a possible linkage of serum UA to plasma BNP and LVEF by these surrounding factors, a single regression analysis was performed (also see Table 2), and the respective effects of age, BMI or Cr on serum UA or plasma BNP are presented in Fig. 2(a-1–d-3). As a result, female gender was positively associated with LVEF (a-1) and Log BNP (a-2) but negatively associated with serum UA (a-3; *P* < 0.001). Age was positively associated with LVEF (b-1) and Log BNP (b-2) but negatively associated with high UA (b-3; *P* < 0.001). BMI was negatively associated with Log BNP (c-2) but positively associated with high UA (c-3) and LVEF (c-1; *P* < 0.001). Additionally, Cr was positively associated with Log BNP (d-2) and serum UA (d-3) but negatively associated with LVEF (d-1; *P* < 0.001). These analyses suggested that surrounding factors such as ageing, BMI and Cr would seemingly modulate the authentic relationship among serum UA, plasma BNP and LVEF.

**Table 2:** Results of Pearson’s product-moment correlation coefficient analysis of hemodynamic parameters.

	Age	BMI	Creatinine	UA	Log BNP	LVEF
Age	—	−0.245	0.122	−0.094	0.348	0.097
		*P* < 0.001	*P* < 0.001	*P* < 0.001	*P* < 0.001	*P* < 0.001
BMI	−0.245	—	0.058	0.178	−0.200	0.034
	*P* < 0.001		*P* = 0.002	*P* < 0.001	*P* < 0.001	*P* = 0.136
Creatinine	0.122	0.058	—	0.248	0.261	−0.169
	*P* < 0.001	*P* = 0.002		*P* < 0.001	*P* < 0.001	*P* < 0.001
UA	−0.094	0.178	0.248	—	0.112	−0.244
	*P* < 0.001	*P* < 0.001	*P* < 0.001		*P* < 0.001	*P* < 0.001
Log BNP	0.348	−0.200	0.261	0.112	—	−0.458
	*P* < 0.001	*P* < 0.001	*P* < 0.001	*P* < 0.001		*P* < 0.001
LVEF	0.097	0.034	−0.169	−0.244	−0.458	—
	*P* < 0.001	*P* = 0.136	*P* < 0.001	*P* < 0.001	*P* < 0.001	

BMI, body mass index; BNP, B-type natriuretic peptide; LVEF, left ventricular ejection fraction; and UA, uric acid.

### Multivariate analysis for determination of the risk factors of LVEF or BNP

Next, to examine the contribution of serum UA and the surrounding factors to LVEF, we adopted a multiple linear regression analysis, as shown in Table 3. As a result, the analysis revealed that age, UA, Cr and BMI represented risks for a low LVEF (*P* < 0.001), except for male gender. Next, multiple linear regression analysis was adapted for the determination of Log BNP, as shown in Table 3. As a result, the analysis revealed that ageing, female gender, high Cr, low BMI and low LVEF represented causes for a high Log BNP (*P* < 0.001); however, UA did not affect the Log BNP. These results suggested that high UA clearly represented a direct risk for a reduction in LVEF, even under the influence of surrounding factors. On the other hand, the association between high UA and plasma BNP was low or unrecognizable.

**Table 3:** Results of the multiple regression analysis to identify clinical factors influencing the LVEF and logarithmic BNP levels (in upper and bottom tables, respectively).

Dependent variable: LVEF (R^2^ = 0.089)	Regression coefficient [95% CI]	Standardized regression coefficient	*P* value	VIF
Age	0.117 [0.071, 0.163]	0.116	<0.001	1.133
Gender	−0.444 [−1.846, 0.957]	−0.015	0.534	1.156
UA	−1.719 [−2.082, −1.356]	−0.218	<0.001	1.160
Creatinine	−5.419 [−7.408, −3.431]	−0.125	<0.001	1.161
BMI	0.380 [0.245, 0.515]	0.130	<0.001	1.156
Constant	55.315 [50.080, 60.550]	—	<0.001	—
Dependent variable: Log BNP (R^2^ = 0.401)	Regression coefficient [95% CI]	Standardized regression coefficient	*P* value	VIF
Age	0.016 [0.014, 0.017]	0.320	<0.001	1.148
Gender	−0.263 [−0.318, −0.209]	−0.180	<0.001	1.156
UA	0.011 [−0.003, 0.026]	0.030	0.122	1.210
Creatinine	0.234 [0.156, 0.312]	0.113	<0.001	1.178
BMI	−0.013 [−0.019, −0.008]	−0.095	<0.001	1.173
LVEF	−0.023 [−0.025, −0.021]	−0.476	<0.001	1.101
Constant	2.198 [1.972, 2.424]	—	<0.001	-

R^2^ adjusted coefficient of determination; BMI, body mass index; BNP, B-type natriuretic peptide; LVEF, left ventricular ejection fraction; UA, uric acid; CI, confidence interval; VIF, variance inflation factor.

### Subgroup analysis using covariance structure analysis

These respective multivariate analyses would still be inadequate for correctly interpreting the risk factors because the factors are expected to confound each other (Fig. 3). We thought that the authentic relationship of high UA with plasma BNP and LVEF was still unclear. To examine the more precise relationship among serum UA, plasma BNP and LVEF, we adopted a covariance structure analysis in the respective subgroups; the subgroups were designed by gender difference and by quartile groups of ageing, BMI and Cr. The covariance structure analysis was planned to construct a simultaneous comparison of the effect of serum UA on plasma BNP and LVEF in one equation model. As a matter of logic, the theoretical path model was proposed by positioning the serum UA level for the determination of both LVEF and Log BNP with a possible causal link from LVEF to Log BNP.

### Results of path models A-1 and A-2 depending on the gender difference

Path models A-1 and A-2 in Fig. 3(a-1,a-2) and Supplementary Table S1 show the results for gender difference. In the female group (path model A-1), the analysis revealed that UA was significantly linked to LVEF and Log BNP (*P* < 0.001). Additionally, there was a significant linkage from LVEF to Log BNP (*P* < 0.001). On the other hand, in the male group (path model A-2), the analysis revealed that UA was significantly linked to LVEF (*P* < 0.001) but not to Log BNP (*P* = 0.423), although there was a significant linkage from LVEF to Log BNP (*P* < 0.001). These results showed that high UA had a negative impact on LVEF in different genders, but it had a positive impact on Log BNP only in females.

### Results of path models B-1, B-2, B-3 and B-4 depending on ageing

**Figure 4:**
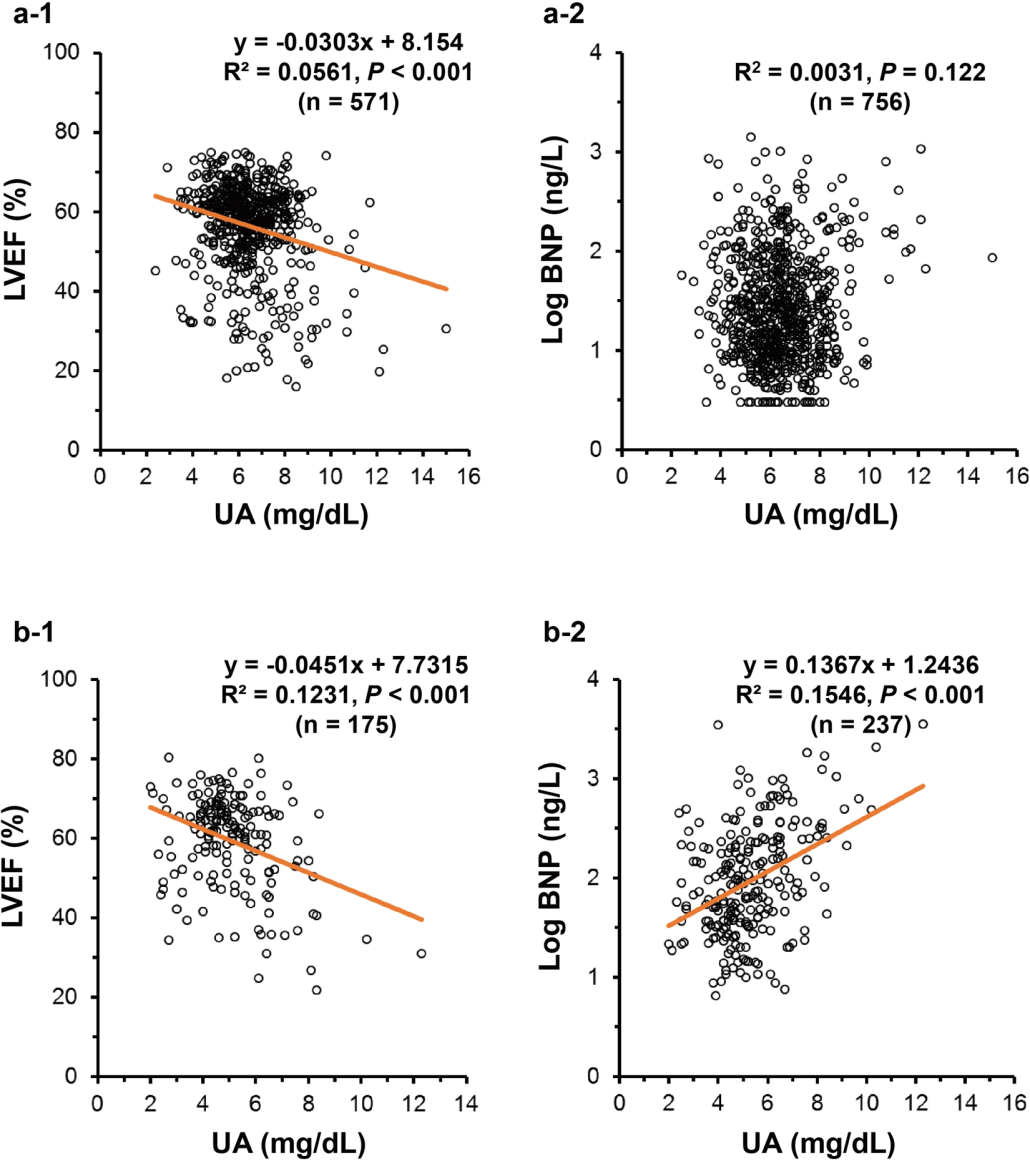
Single regression analysis between UA and LVEF and between UA and Log BNP among obese, adolescent males, and among lean, aged females These upper and lower panels demonstrate a single regression analysis between UA and LVEF, and UA and Log BNP among each group, divided into obese, adolescent males (upper panels: age <67 years and BMI ≥ 24.2 kg/m^2^) and lean, aged females (lower panels: age ≥67 years and BMI < 24.2 kg/m^2^). Scatter plots and a simple regression line (orange straight line) with the regression equation demonstrate the correlation between the two values among UA, plasma BNP, and LVEF. BNP, B-type natriuretic peptide; LVEF, left ventricular ejection fraction; UA, uric acid.

Path model B-1, B-2, B-3 and B-4 in Fig. 3(b-1,b-2,b-3 and b-4) and Supplementary Table S2 show the results for ageing. The analysis in path model B-1 showed that UA was significantly linked to LVEF (*P* < 0.001) but not to Log BNP (*P* = 0.221). Path model B-2 showed that serum UA showed a significant link to LVEF (*P* < 0.001) and an inverse link to Log BNP (not a positive link) (*P* = 0.015). Path model B-3 showed that serum UA had a significant link to LVEF (*P* < 0.001) and Log BNP (*P* = 0.002). Additionally, path model B-4 showed that serum UA had a significant link to LVEF (*P* < 0.001) and Log BNP (*P* = 0.005). These results showed that high UA had a significant impact on LVEF at any age and on Log BNP only in the elderly population (over 67 years).

### Results of path models C-1, C-2, C-3 and C-4 depending on BMI

Path models C-1, C-2, C-3 and C-4 in Fig. 3(c-1,c-2,c-3 and c-4) and Supplementary Table S3 show the results for BMI. The analysis in path model C-1 revealed that serum UA was significantly linked to LVEF (*P* < 0.001) and Log BNP (*P* = 0.010). Path model C-2 showed that serum UA showed a significant link to LVEF (*P* < 0.001) but not to Log BNP (*P* = 0.828). Path model C-3 showed that serum UA had a significant link to LVEF (*P* < 0.001) but not to Log BNP (*P* = 0.532). Additionally, path model C-4 showed that serum UA had a significant link to LVEF (*P* < 0.001) but not to Log BNP (*P* = 0.461). These results showed that high UA had a significant impact on LVEF at any stage of BMI, but it had an effect on Log BNP only in the lean body mass population (BMI ≤ 21.8).

### Results of path models D-1, D-2, D-3 and D-4 depending on Cr

Path models D-1, D-2, D-3 and D-4 in Fig. 3(d-1,d-2,d-3 and d-4) and Supplementary Table S4 show the results for Cr. The analysis of path model D-1 revealed that serum UA was not significantly associated with LVEF (*P* = 0.303) and was significantly associated with Log BNP (not a positive link) (*P* = 0.008). Path model D-3 showed that serum UA was significantly associated with LVEF (*P* < 0.001) but not with Log BNP (*P* = 0.858). Additionally, path model D-4 showed that serum UA was significantly associated with LVEF (*P* < 0.001) but not with Log BNP (*P* = 0.857).

### Summary of the results of the covariance structure analysis and another single regression analysis among the discriminative groups

Summarizing the results of the covariance structure analysis among all path models (A, B, C and D in Fig. 3) revealed that high serum UA was almost always associated with reduced LVEF, except in one model (path model D-1 in Fig. 3). On the other hand, the authentic association between serum UA and plasma BNP was recognized only among several subgroups: the female gender group (path model A-1 in Fig. 3), the aged groups (path models B-3 and B-4 in Fig. 3) and the lean body mass group (path model C-1 in Fig. 3). When taken together, the results showed that the association of high serum UA and high plasma BNP might be clarified more sharply among lean aged females but was not observed among obese, adolescent males. In the final analysis, a single regression analysis was performed in respective population groups with cut-off values of 67 years in age and 24.2 in BMI, as shown in Fig. 4. As a consequence, there was indeed a close correlation between serum UA and LVEF (b-1 in Fig. 4) and between serum UA and Log BNP (b-2 in Fig. 4, *P* < 0.001) among lean aged females. On the other hand, there was a close linkage between serum UA and LVEF (a-1 in Fig. 4, *P* < 0.001) but never between serum UA and Log BNP (a-2 in Fig. 4, *P* = 0.122) among obese, adolescent males.

## Discussion

This study was planned to revalidate the harmful effects of high serum UA on LV function from the perspective of plasma BNP, which is regarded as a sensitive biomarker of LV dysfunction. Thus, the precise relationship among serum UA, plasma BNP and LVEF was examined in a large number of patients with cardiovascular diseases. First, we performed a multivariate analysis, which showed that high serum UA was a significant cause of reduced LVEF but did not affect plasma BNP. This result represents a curious consequence because serum UA, LVEF and plasma BNP were closely linked in the single regression analysis. We then hypothesized that some surrounding factors mediate high serum UA and high plasma BNP, blunting their authentic relationship. By reference to the single regression analysis including surrounding factors, we proposed hierarchical equation models in the respective subgroups. Importantly, the models clearly demonstrated that 1) high serum UA was sharply associated with reduced LVEF and that 2) plasma BNP authentically responded to high serum UA, but only under the appropriate conditions, such as among lean aged females but never among obese, adolescent males.

As observed above, we reaffirmed that high serum UA is a risk factor for cardiac dysfunction, which can be evaluated not only by LVEF but also by plasma BNP, even under some limitations. Although the molecular mechanisms underlying the unfavorable effects of high serum UA on the heart have not been precisely examined, potential mechanisms are as follows. The UA level is elevated with subsequent upregulation of the xanthine oxidase (XO) enzymatic pathway, which accounts for the production of reactive oxygen species (ROS) via nicotinamide adenine dinucleotide phosphate (NADPH)^26–28^. High XO-induced ROS production would be significant throughout the whole body, but the precise mechanism in the local heart has not been well clarified. However, it has recently been reported that cardiac function is reduced in accordance with energy deficiency, which is linked to possible cardiac UA production and XO activity^29–31^. If XO enzymatic activity is sufficiently upregulated for a local increase in oxidative stress in the heart, BNP production may also be activated under such circumstances^32^. As outlined above, high serum UA, high plasma BNP and low LVEF would be mutually related in heart failure by oxidative stress, which is probably activated not only throughout the whole body but also locally in the heart.

The possible reasons or mechanisms for blunting the close linkage between serum UA and plasma BNP under a given set of conditions are as follows. First, we briefly describe the possible reasons for the beclouded linkage between serum UA and plasma BNP from the perspective of obesity. The molecular mechanisms have just started to be examined; however, it has recently been reported that natriuretic peptide reduces body mass by improving the biological function of adipocytes and accelerating fat burning^33^. On the other hand, free fatty acid suppresses the production of BNP^34,35^. These pathological actions would lead to a close linkage between low fat content and high plasma BNP or between high fat content and low plasma BNP. However, it is quite natural that high body mass would be substantially linked to high UA, suggesting that adipose tissue or FFA has different effects on serum UA (raising) and plasma BNP (lowering). Thus, the authentic correlation between serum UA and plasma BNP becomes less visible in obesity.

Second, it is well accepted that serum UA is higher in youth because of increased metabolic activity, high physical activity and a high level of food intake without a relationship to heart failure status. Additionally, the plasma BNP level would generally be low in the young population compared with the aged population^24^. Ageing then likely has different effects on serum UA (raising) and plasma BNP (lowering); thus, the authentic correlation between serum UA and plasma BNP becomes less apparent in the adolescent population.

Third, the strong linkage was absolute between serum UA and plasma BNP in females; however, the linkage disappeared in males. It is difficult to proceed with a discussion of the precise mechanism(s). Sex steroid hormones may be associated with serum UA^36–38^ and with plasma BNP via a wide variety of mechanisms^39–41^.

Finally, a close linkage between serum UA and plasma BNP was hardly observed in any stage of renal dysfunction. The reasons or mechanisms are unknown; however, other risk factors including ageing, male gender and obesity, which are highly correlated with renal dysfunction, would have substantially affected the values of UA and BNP in diverse ways. Importantly, the close correlation between serum UA and reduced LVEF was mostly recognizable in advanced stages of renal dysfunction in this study.

The direct effect of high serum UA on cardiac damage would be definite based on the results of the current and our previous study^14^. However, this study clearly demonstrated that the adverse effect of high serum UA on heart failure status would likely be underestimated only by measuring plasma BNP, which must be dealt with carefully in clinical practice. It is important to search for factors or clinical parameters that are strongly associated with serum UA. For example, oxidative stress markers may be conceivable. Additionally, the direct measurement of XO activity in the circulation enters the picture. On the other hand, the harmful effects of high serum UA are almost always demonstrated by the observed cardiac function such as the LVEF. In a further study, we should investigate whether high serum UA is more likely linked to patients with heart failure with an reduced ejection fraction (HFrEF) or to those with heart failure with a preserved ejection fraction (HFpEF).

### Limitations of this study

This was a cross-sectional study, and regarding the underlying diseases of the patients in this study, most had ischemic heart disease (84.2%). Further analysis is warranted to determine the adverse effect of high UA prospectively in a large number and various kinds of patients with heart failure. Additionally, in the present study, we did not directly examine the biological interaction between BNP and UA. Therefore, there may be a limitation to determining whether UA affects BNP or whether BNP affects UA as a definitive conclusion. A precise biological analysis should be conducted in the future.

## Conclusion

High serum UA is a direct risk factor for cardiac dysfunction from the perspective of plasma BNP; however, augmentation of plasma BNP in response to high serum UA would likely be restricted among obese adolescent males. On the other hand, the observed LV systolic dysfunction such as LVEF sharply reflects high serum UA on an almost constant basis.

## Methods

### Study patients

The study patients consisted of 3,077 subjects with any cause who were consecutively admitted to our institutions from 2012 to 2016. All patients underwent cardiac catheterization for an evaluation of respective cardiac disorders. We excluded patients receiving hemodialysis because cardiac function was largely changed by artificial volume control. The ethics committee of The Jikei University School of Medicine approved the study protocol (24-355[7121]), and we complied with our institution’s routine ethical regulations. Informed consent was obtained from each patient, and all clinical investigations were conducted in accordance with the principles expressed in the Declaration of Helsinki. According to our routine ethical regulations, we posted a notice about the study design and contact information at a public location in our institution.

### Underlying cardiac diseases and their definitions

Baseline diseases, hypertension, diabetes mellitus, and dyslipidemia were defined as previously described^23,24^. The definitions of the diseases were as follows: In brief, ischemic heart disease (IHD) was diagnosed by symptoms, electrocardiography results, blood sampling, and the morphology of the coronary arteries. The patients with IHD included those with clinically stable IHD. Organic stenosis was defined as ≥75% occlusion of the coronary arteries on coronary angiography. The patients with coronary spastic angina were included in the IHD group if the disease activity was stable, and a provocation test was planned during hospitalization. Valvular diseases included heart failure caused by moderate valvular disease and patients who were scheduled for surgery. Arrhythmia included the need for catheter ablation, an implantable cardioverter-defibrillator, cardiac resynchronization therapy, and patients with a pacemaker or syncope. Cardiomyopathy was defined when patients were diagnosed before admission and underwent treatment or if they were diagnosed after admission (excluding cases of ischemic cardiomyopathy). Infectious heart disease included pericarditis, myocarditis, and infectious endocarditis. BMI was calculated by the square of the height divided by the weight on admission.

### Cardiac catheterization

LVEF was obtained from the left ventriculography (LVG) trace during the end-systolic and end-diastolic phases. The contrast LVG images were acquired at a frame rate of 30 frames per second in the right anterior oblique 30-degree projection. The LVEF was calculated from single-plane cineangiograms by means of the area-length formula using a semi-automated trace method with QAngio XA version 7.1 (Medis Medical Imaging Systems B.V., Leiden, The Netherlands).

### Blood sampling and measurement of plasma BNP and other levels

We collected data for plasma BNP and other levels during cardiac catheterization. Whole blood (5 mL) was collected in tubes containing sodium ethylenediaminetetraacetic acid (EDTA) (1 mg/mL blood). Plasma BNP was measured with a rapid enzyme-linked immunosorbent assay (non-extracted) using an antibody against human BNP (Shionogi Co. Ltd., Tokyo, Japan). Serum biochemical analyses, including UA and Cr, were performed in a central laboratory in our hospital during the study.

### Statistical analysis

Continuous variables are expressed as the means ± standard deviation (SD) or medians with the interquartile range [IQR]. Categorical variables are expressed as the percentages of overall and/or each sub-group and were compared using a chi-square test as appropriate. Comparisons between two data sets of continuous variables were performed using Pearson’s product-moment correlation coefficient analysis, and significant differences between them were analyzed using a *t*-test and/or the Mann–Whitney *U* test where appropriate. After confirming the normality of distribution of the BNP variable by using a formal test, to achieve a normal distribution, the BNP value was log-transformed to (Log) BNP before conducting the analysis. Multiple regression analysis was performed when multiple values were compared. All statistical analyses were performed using SPSS Statistics version 23.0 (SPSS Inc., Chicago, IL, USA).

Path analysis based on covariance structure analysis was used to investigate the relationship among clinical factors in this study population and, in particular, to identify probable causal effects in heart failure. Path analysis was performed using IBM SPSS AMOS version 23 (Amos Development Corporation, Meadville, PA, USA). The obtained structural equation models were tested and confirmed at the significance level of *P* values < 0.05. The implementation procedures for the covariance structure analysis have been described previously^14,42–47^. The paths between the variables were drawn from the independent to the dependent variables with directional arrows that were used for every regression model (i.e., arrowhead at one end only). A two-way arrow between two variables indicated a correlation between these variables. For every regression, the total variance in the dependent variable was theorized to be caused either by the independent variables in the model or by extraneous variables (e). Each path had a coefficient that indicated the standardized coefficient of the regressing independent variable on the dependent variable of the relevant path. The indirect effect was determined by multiplying the path coefficients of the intervening variables. The causality model defined certain hierarchical regression models that compare clinical factors in LVEF and plasma BNP. The corresponding author had full access to all the data in the study and takes responsibility for both its integrity and the data analysis.

## Supplementary information

Supplementary Tables S1-S4

## Data Availability

All relevant or analyzed data during this study are included in this published article (and its Supplementary Table file).
