# Supplementary material for: High Serum Uric Acid is Highly Associated with a Reduced Left Ventricular Ejection Fraction Rather than Increased Plasma B-type Natriuretic Peptide in Patients with Cardiovascular Diseases
Source: Sci Rep. 2019 Jan 24;9:682. doi: 10.1038/s41598-018-37053-0 (PMC6346056; doi:10.1038/s41598-018-37053-0)
Supplement: Supplementary file 1 — Supplementary Tables S1-S4 [file 41598_2018_37053_MOESM1_ESM.pdf]

Supplementary Table

High Serum Uric Acid Is Highly Associated with a Reduced Left Ventricular Ejection Fraction Rather than Increased Plasma B-type Natriuretic Peptide in Patients with Cardiovascular Diseases

Yoshitsugu Oki, MD, Makoto Kawai, MD, PhD, Kosuke Minai, MD, PhD, Kazuo Ogawa, MD, PhD, Yasunori Inoue, MD, PhD, Satoshi Morimoto, MD, PhD, Toshikazu Tanaka, MD, PhD, Tomohisa Nagoshi, MD, PhD, Takayuki Ogawa, MD, PhD, and Michihiro Yoshimura, MD, PhD.

Division of Cardiology, Department of Internal Medicine, The Jikei University School of Medicine

Corresponding author: Makoto Kawai, M.D., Ph.D.

3-25-8 Nishi-Shimbashi, Minato-ku, Tokyo 105-8461, Japan.

Telephone: +81-3-3433-1111, Facsimile: +81-3-3459-6043

E-mail: cadmk@jikei.ac.jp

Supplemental Table S1. Results of the path models A-1 and A-2

Gender			Female			Male		
Path model			A-1 (n = 542)			A-2 (n = 2,535)		
Clinical Factor			Regression coefficient (standardized)					
			Direct	Indirect	Total	Direct	Indirect	Total
Log BNP	←	UA	0.202	0.088	0.290	-0.016	0.129	0.113
		P value	<0.001			0.423		
Log BNP	←	LVEF	-0.366	0	-0.366	-0.521	0	-0.521
		P value	<0.001			<0.001		
		R^2	0.210			0.267		
LVEF	←	UA	-0.240	0	-0.240	-0.248	0	-0.248
		P value	<0.001			<0.001		
		R^2	0.058			0.062		

The results (direct, indirect, and total effects) of the path model theoretical proposed analysis to identify the clinical factors influencing each other (see a-1 and a-2 in Figure 3).

BMI, body mass index; BNP, B-type natriuretic peptide; LVEF, left ventricular ejection fraction; UA, uric acid. R^2: squared multiple correlations.

Supplemental Table S2. Results of the path models B-1, B-2, B-3, and B-4

Quartile group			Q1 Age ≤58 years			Q2 Age 59-66 years			Q3 Age 67-74 years			Q4 Age ≥75 years		
Path model			B-1 (n = 752)			B-2 (n = 765)			B-3 (n = 771)			B-4 (n = 789)		
Clinical Factor			Regression coefficient (standardized)											
			Direct	Indirect	Total	Direct	Indirect	Total	Direct	Indirect	Total	Direct	Indirect	Total
Log BNP	←	UA	-0.043	0.191	0.148	-0.086	0.115	0.029	0.110	0.143	0.252	0.104	0.081	0.185
		P value	0.221			0.015			0.002			0.005		
Log BNP	←	LVEF	-0.612	0	-0.612	-0.542	0	-0.542	-0.535	0	-0.535	-0.409	0	-0.409
		P value	<0.001			<0.001			<0.001			<0.001		
		R^2	0.360			0.281			0.330			0.195		
LVEF	←	UA	-0.311	0	-0.311	-0.212	0	-0.212	-0.266	0	-0.266	-0.197	0	-0.197
		P value	<0.001			<0.001			<0.001			<0.001		
		R^2	0.097			0.045			0.071			0.039		

The results (direct, indirect, and total effects) of the path model theoretical proposed analysis to identify the clinical factors influencing each other (see b-1, b-2, b-3 and b-4 in Figure 3).

BMI, body mass index; BNP, B-type natriuretic peptide; LVEF, left ventricular ejection fraction; UA, uric acid. Q: quartile, R^2: squared multiple correlations.

Supplemental Table S3. Results of the path models C-1, C-2, C-3, and C-4

Quartile group			Q1 BMI ≤21.8 kg/m^2			Q2 BMI 21.9-24.1 kg/m^2			Q3 BMI 24.2-26.4 kg/m^2			Q4 BMI ≥26.5 kg/m^2		
Path model			C-1 (n = 765)			C-2 (n = 760)			C-3 (n = 765)			C-4 (n = 787)		
Clinical Factor			Regression coefficient (Standardized)											
			Direct	Indirect	Total	Direct	Indirect	Total	Direct	Indirect	Total	Direct	Indirect	Total
Log BNP	←	UA	0.099	0.134	0.287	-0.008	0.104	0.096	0.023	0.123	0.146	-0.027	0.077	0.050
		P value	0.010			0.828			0.532			0.461		
Log BNP	←	LVEF	-0.473	0	-0.473	-0.446	0	-0.446	-0.496	0	-0.496	-0.431	0	-0.431
		P value	<0.001			<0.001			<0.001			<0.001		
		R^2	0.271			0.197			0.252			0.182		
LVEF	←	UA	-0.396	0	-0.396	-0.233	0	-0.233	-0.248	0	-0.248	-0.179	0	-0.179
		P value	<0.001			<0.001			<0.001			<0.001		
		R^2	0.157			0.054			0.062			0.032		

The results (direct, indirect, and total effects) of the path model theoretical proposed analysis to identify the clinical factors influencing each other (see c-1, c-2, c-3 and c-4 in Figure 3).

BMI, body mass index; BNP, B-type natriuretic peptide; LVEF, left ventricular ejection fraction; UA, uric acid. Q: quartile, R^2: squared multiple correlations.

Supplemental Table S4. Results of the path models D-1, D-2, D-3, and D-4

Quartile group			Q1 Cr ≤0.71 mg /dL			Q2 Cr 0.72-0.83 mg/dL			Q3 Cr 0.84-0.97 mg/dL			Q4 Cr ≥0.98 mg/dL		
Path model			D-1 (n = 669)			D-2 (n = 690)			D-3 (n = 688)			D-4 (n = 698)		
Clinical Factor			Regression coefficient (Standardized)											
			Direct	Indirect	Total	Direct	Indirect	Total	Direct	Indirect	Total	Direct	Indirect	Total
Log BNP	←	UA	-0.097	0.017	-0.080	-0.076	0.097	0.021	-0.006	0.125	0.118	-0.007	0.110	0.103
		P value	0.008			0.040			0.858			0.857		
Log BNP	←	LVEF	-0.386	0	-0.386	-0.423	0	-0.423	-0.503	0	-0.503	-0.457	0	-0.457
		P value	<0.001			<0.001			<0.001			<0.001		
		R^2	0.155			0.170			0.251			0.207		
LVEF	←	UA	-0.044	0	-0.044	-0.229	0	-0.226	-0.248	0	-0.248	-0.240	0	-0.240
		P value	0.303			<0.001			<0.001			<0.001		
		R^2	0.002			0.053			0.062			0.058		

The results (direct, indirect, and total effects) of the path model theoretical proposed analysis to identify the clinical factors influencing each other (see d-1, d-2, d-3 and d-4 in Figure 3).

BMI, body mass index; BNP, B-type natriuretic peptide; Cr, Creatinine; LVEF, left ventricular ejection fraction; UA, uric acid. Q: quartile, R^2: squared multiple correlations.
